# Laquinimod, a Quinoline-3-Carboxamide, Induces Type II Myeloid Cells That Modulate Central Nervous System Autoimmunity

**DOI:** 10.1371/journal.pone.0033797

**Published:** 2012-03-30

**Authors:** Ulf Schulze-Topphoff, Aparna Shetty, Michel Varrin-Doyer, Nicolas Molnarfi, Sharon A. Sagan, Raymond A. Sobel, Patricia A. Nelson, Scott S. Zamvil

**Affiliations:** 1 Department of Neurology and Program in Immunology, University of California, San Francisco, San Francisco, California, United States of America; 2 Department of Pathology, Stanford University, Stanford, California, United States of America; Klinikum rechts der Isar der Technischen Universitaet Muenchen, Germany

## Abstract

Laquinimod is a novel oral drug that is currently being evaluated for the treatment of relapsing-remitting (RR) multiple sclerosis (MS). Using the animal model for multiple sclerosis, experimental autoimmune encephalomyelitis (EAE), we examined how laquinimod promotes immune modulation. Oral laquinimod treatment reversed established RR-EAE and was associated with reduced central nervous system (CNS) inflammation, decreased Th1 and Th17 responses, and an increase in regulatory T cells (Treg). In vivo laquinimod treatment inhibited donor myelin-specific T cells from transferring EAE to naive recipient mice. In vivo laquinimod treatment altered subpopulations of myeloid antigen presenting cells (APC) that included a decrease in CD11c^+^CD11b^+^CD4^+^ dendritic cells (DC) and an elevation of CD11b^hi^Gr1^hi^ monocytes. CD11b^+^ cells from these mice exhibited an anti-inflammatory type II phenotype characterized by reduced STAT1 phosphorylation, decreased production of IL-6, IL-12/23 and TNF, and increased IL-10. In adoptive transfer, donor type II monocytes from laquinimod-treated mice suppressed clinical and histologic disease in recipients with established EAE. As effects were observed in both APC and T cell compartments, we examined whether T cell immune modulation occurred as a direct effect of laquinimod on T cells, or as a consequence of altered APC function. Inhibition of Th1 and Th17 differentiation was observed only when type II monocytes or DC from laquinimod-treated mice were used as APC, regardless of whether myelin-specific T cells were obtained from laquinimod-treated or untreated mice. Thus, laquinimod modulates adaptive T cell immune responses via its effects on cells of the innate immune system, and may not influence T cells directly.

## Introduction

Laquinimod (N-ethyl-N-phenyl-5-chloro-1, 2-dihydroxy-1-methyl-2-oxo-quinoline-3-carboxamide) is a novel oral agent with immunomodulatory properties that is currently under evaluation for treatment of relapsing-remitting (RR) multiple sclerosis (MS) and other autoimmune diseases [1]–[3]. Laquinimod is structurally related to roquinimex (linomide), which demonstrated efficacy in MS [4], although its development was halted after unanticipated serious adverse events occurred in a phase III trial [5]. In screening a large number of chemically modified quinoline-3-carboxamides, laquinimod was discovered to have less toxicity and greater efficacy than linomide in the MS model, experimental autoimmune encephalomyelitis (EAE) [6]. Laquinimod has since shown efficacy in phase II and phase III MS clinical trials, without evident immunosuppression or significant toxicities [1], [2], [7].

Studies in EAE indicate that laquinimod can promote immune modulation and neuroprotection [8], [9]. Laquinimod inhibited development of EAE [9]–[11] and suppressed production of proinflammatory cytokines [8], [9], [12]. However, those studies did not address the mechanisms responsible for alteration of T cell responses. It is possible that laquinimod could act directly on T cells, or modulate T cell responses through its effects on accessory cells. In this regard, it is now understood that some medications currently used in MS treatment exert effects through antigen presenting cells (APC) [13], [14], which then contribute to T cell immune modulation [13], [15].

In this study, we investigated laquinimod's mechanism of action for immune modulation. Oral laquinimod treatment initiated during remission prevented further relapses and reduced central nervous system (CNS) inflammation. In vivo laquinimod treatment was associated with reduced proinflammatory Th1 and Th17 responses, elevation of CD4^+^CD25^+^Foxp3^+^ regulatory T cells (Treg), and alterations in dendritic cells (DC) and monocyte subpopulations. These myeloid cells exhibited an anti-inflammatory (type II) cytokine and signaling profile [16], [17]. When used as APC, they promoted development of Treg and inhibited differentiation of proinflammatory T cells, regardless of whether or not T cells were exposed to laquinimod. Our results demonstrate that laquinimod modulates T cell immune responses through a direct effect on myeloid APC.

## Results

### Laquinimod reverses EAE and inhibits pathogenic T cell immune responses

**Figure 1:**
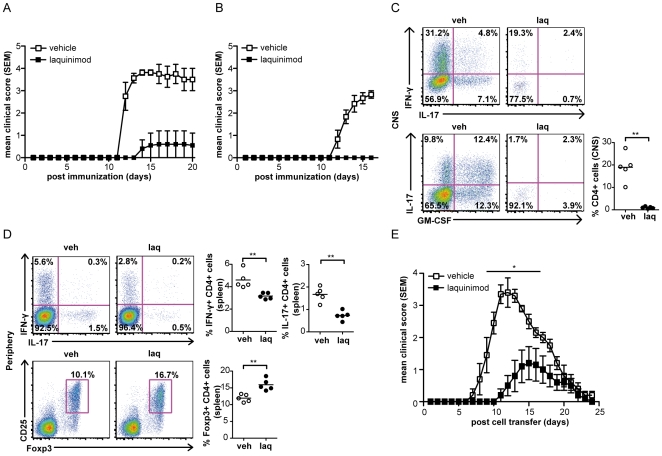
Laquinimod prevents EAE and decreases encephalitogenicity of T cells. DBA/1 (A) mice and C57BL/6 mice (B) were treated daily with laquinimod (25 mg/kg, n = 6/group) or vehicle (water, n = 6/group) starting from the day of immunization with rMOG 1-125 (DBA/1) or MOG p35-55 (C57BL/6). Fourteen days after immunization, CNS infiltrating cells (C) and spleen cells (D) from C57BL/6 mice treated with laquinimod or vehicle were evaluated for secretion of IFN-γ, IL-17, GM-CSF and for expression of CD25 and Foxp3 by CD4^+^ cells. Representative FACS staining is shown including quantification (n = 4–5 mice per group). (E) Laquinimod decreases pathogenic potential of T cells. Splenocytes and lymph node cells were isolated from PLP-immunized mice treated with laquinimod or vehicle. Cells were restimulated in vitro, and 10^7^ cells were transferred into naïve SJL/J recipients (n = 5/group). Data are representative of two independent experiments. For EAE disease course, mean disease score ± s.e.m. are displayed; for other experiments mean ± s.d. *P<0.05, **P<0.01, Mann-Whitney U test.

Laquinimod was tested in prevention of chronic EAE in two mouse strains, DBA/1 (H-2^q^) and C57BL/6 (H-2^b^). When immunized with recombinant MOG 1–125, DBA/1 mice are known to develop a severe disease course [18]. As shown in Figure 1A, oral laquinimod treatment prevented development of EAE in DBA/1 mice. Similarly, oral laquinimod treatment prevented induction of MOG p35-55-induced EAE in C57BL/6 mice.(Fig. 1B). Very few infiltrating CD4^+^ T cells were detected in the CNS of laquinimod-treated mice, whereas abundant CNS infiltration of CD4^+^ Th1, Th17 and GM-CSF expressing cells were detected in vehicle-treated mice (Fig. 1C, p<0.01). Recent studies demonstrated the important role of GM-CSF on the encephalitogenicity of Th1 and Th17 cells in EAE [19], [20]. To determine whether laquinimod prevents EAE by modulating peripheral T cell immune responses important for the development of the disease, CD4^+^ splenocytes from mice treated with laquinimod or vehicle were analyzed for their inflammatory profile. Preventive treatment with laquinimod significantly decreased the number of IFN-γ and IL-17 producing cells (Fig. 1D, p<0.01). In addition to a decrease in Th1 and Th17 responses, we observed a corresponding increase in CD4^+^CD25^+^Foxp3-expressing regulatory T cells (Treg, Fig. 1D, p<0.01). As we observed that laquinimod treatment inhibited proinflammatory T cell responses, we addressed whether it altered the encephalitogenic potential of myelin-specific T cells when transferred to naïve mice. As shown in Figure 1E, PLP-specific T cells from laquinimod-treated mice, which produced reduced quantities of proinflammatory cytokines, were less encephalitogenic than T cells from vehicle-treated mice (Fig. 1E, Fig. S1).

**Figure 2:**
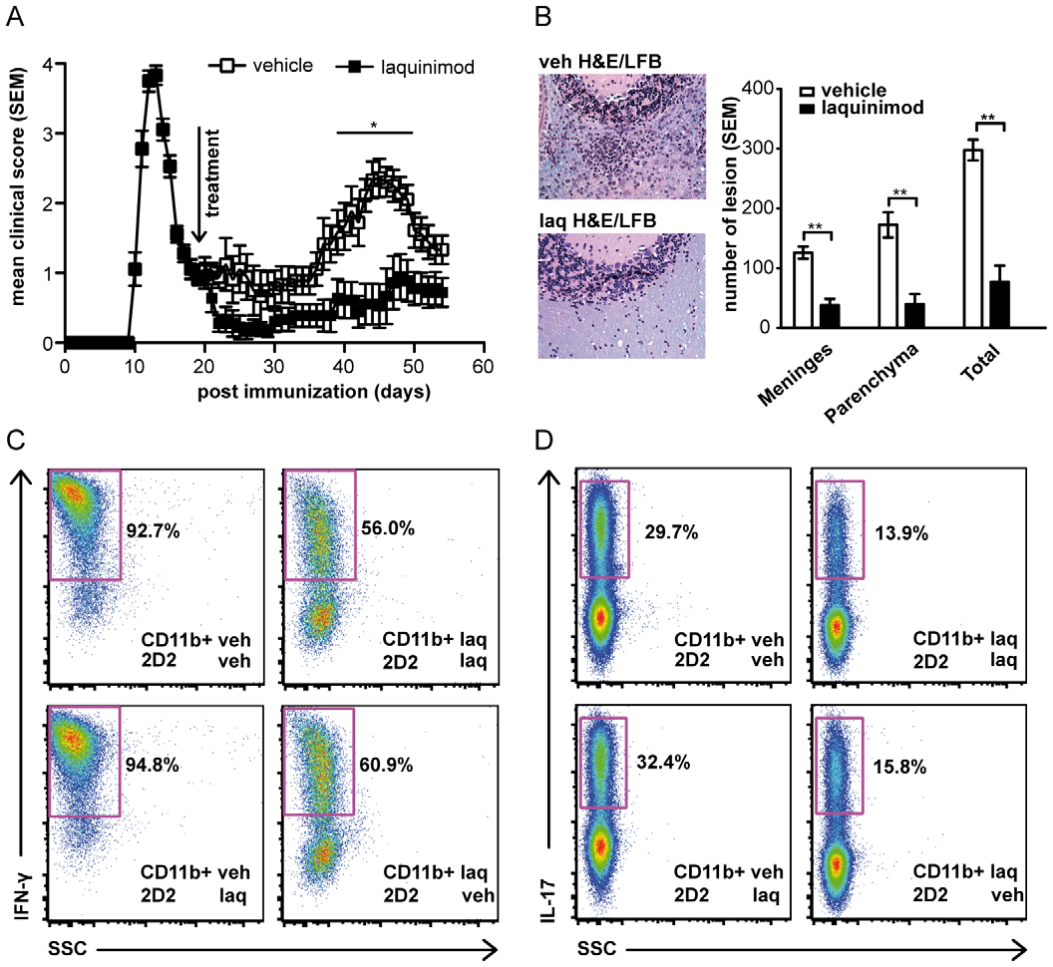
Laquinimod reverses RR-EAE and inhibits inflammatory T cell responses via a direct effect on myeloid APC. (A) Daily oral laquinimod treatment reverses relapsing remitting EAE. SJL/J mice were immunized with PLP p139-151 and treated with laquinimod or vehicle (n = 9) at the remission phase (arrow points to the start of the treatment). (B) Lesion quantification showed reduced total number of meningeal and parenchymal inflammatory foci in SJL/J mice treated with laquinimod after first exacerbation of the disease. Representative Luxol fast blue-H&E staining of the cerebellum is shown. (C, D) Laquinimod-treated myeloid APC inhibit differentiation of naive T cells into Th1 and Th17 cells. Whole splenic in vivo laquinimod-treated or untreated CD11b^+^ cells were used as APC in co-culture with untreated naive (CD4^+^CD44^−^CD62L^+^) T cells from MOG p35-55 TCR-transgenic mice (2D2). Conversely, naive T cells were isolated from laquinimod- or vehicle-treated 2D2 mice and cultured with purified vehicle-treated CD11b^+^ cells and Ag (MOG p35-55). Polarization of naïve T cells into Th1 lineage was induced by IL-12 (C) and polarization into Th17 lineage was induced by IL-23, IL-6 and TGF-β (D). Intracellular cytokine staining for IFN-γ and IL-17 after three days in culture is shown. For all experiments, data shown are representative of two independent experiments. For EAE disease course and other experiments, mean disease score and mean ± s.e.m. are displayed; *P<0.05, Mann-Whitney U test.

It was demonstrated that laquinimod treatment prior to, or at the time of onset of clinical signs, suppressed EAE development in C57BL/6 mice immunized with MOG p35-55 [9]. In a study of EAE induced by myelin basic protein in B10.RIII mice, it was observed that laquinimod treatment after onset, but before peak of clinical disease, prevented subsequent relapses [21]. We examined whether laquinimod could reverse established RR-EAE in SJL/J mice. When oral laquinimod treatment was initiated during remission after the first exacerbation, it prevented subsequent relapses (Fig. 2A). Histological analysis of these mice revealed a significant reduction in CNS inflammatory lesions (Fig. 2B).

### Laquinimod mediates T cell immune responses via a direct effect on APC function

We next addressed whether laquinimod mediated immune modulation by acting on T cells directly or through alteration of APC function. Purified CD11b^+^ cells from laquinimod- or vehicle-treated mice were tested as APC for stimulation of naïve MOG p35-55-specific (2D2) T cells under Th1- or Th17-polarizing conditions. In a reciprocal manner, we examined how purified naïve MOG p35-55-specific T cells from vehicle- or laquinimod-treated 2D2 mice responded to CD11b^+^ APC from untreated mice. CD11b^+^ APC from laquinimod-treated mice, but not from untreated mice, inhibited the development of Th1 (Fig. 2C) and Th17 cells (Fig. 2D). In contrast, no difference in Th1 and Th17 polarization was observed when purified MOG-specific T cells from laquinimod-treated mice were cultured with untreated CD11b^+^ APC (Fig. 2C, 2D). In line with these findings, proliferation of 2D2 T cells was reduced when CD11b^+^ cells from laquinimod-treated mice were used as APC, although no effect on T cell proliferation was observed when 2D2 T cells from laquinimod-treated mice were cultured with vehicle-treated CD11b^+^ cells or activated by anti-CD3/anti-CD28 in the absence of APC (Fig. S2).

**Figure 3:**
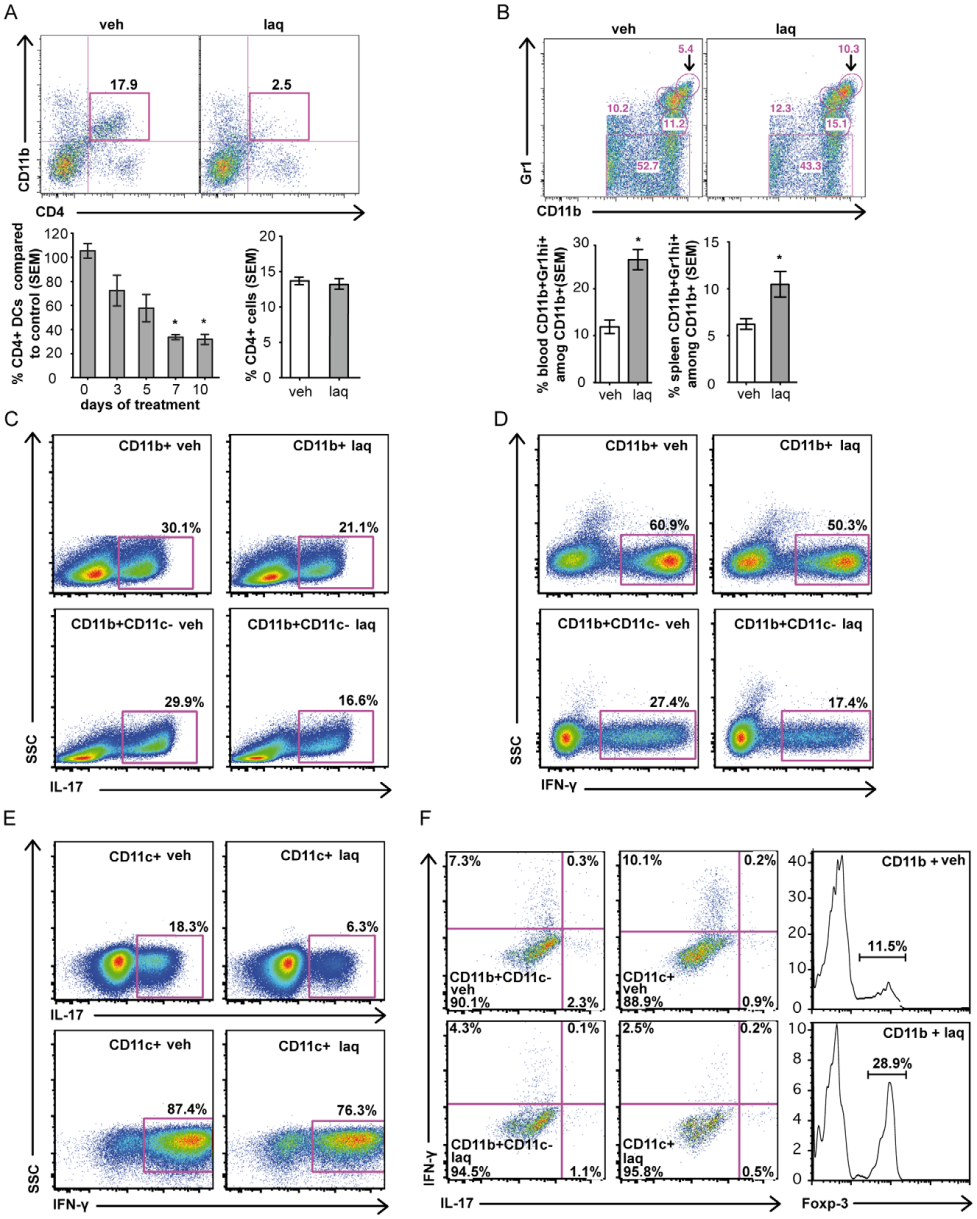
Laquinimod alters myeloid APC subsets and inhibits Th1 and Th17 polarization of myelin-specific T cells. All experiments were conducted with myeloid-APC purified (purity ≥95%) by MACS sorting from mice treated with laquinimod or vehicle for ten days. (A) DC were isolated from the spleen and defined as CD11c^high^. Percentages of splenic CD11c^high^ CD4^+^ cDC (CD4^+^CD11b^+^) from laquinimod-treated or vehicle-treated mice (n = 4), and percentage of total splenic CD4^+^ cells from laquinimod- and vehicle-treated mice (n = 5) are shown. (B) Splenic and blood CD11b^+^ cells were stained for Gr1. Relative percentages of CD11b^+^Gr1^hi^ (arrow points to Gr1^hi^ cells) out of total splenic and blood CD11b^+^ cells from mice treated with laquinimod (dark grey bar) or vehicle (white bar) is shown (n = 4). (C–E) Purified splenic CD11b^+^, CD11b^+^CD11c^−^ (C, D) or CD11c^+^ (E) cells were used as APC in co-culture with naïve CD4^+^ 2D2 T cells and Ag (MOG p35-55). Th1 and Th17 differentiation were induced as described above. Intracellular cytokine staining shows the percentage of IL-17 and IFN-γ after 3 days in culture (gate). (F) Purified splenic CD11b^+^CD11c^−^ or CD11c^+^ cells were used as APC in co-culture with naïve CD4^+^ 2D2 T cells under non-polarizing conditions. FACS analyses after four days in culture for IL-17 and IFN-γ and for Foxp3 by CD4^+^ cells is shown. Data shown as mean ± s.e.m. are representative of at least two independent experiments; *P<0.05, Mann-Whitney U test.

As our data indicated that laquinimod-mediated T cell immune modulation was driven through its influence on APC, we investigated how oral laquinimod treatment affected individual subsets of myeloid APC. First, we examined classical CD11c^high^ dendritic cells (cDC), including CD8^+^ cDC (CD8a^+^ CD4^−^ CD11b^−^) and CD4^+^ cDC (CD4^+^ CD8^−^ CD11b^+^). A significant decrease of CD4^+^ cDC, which are particularly potent APC among subpopulations of DC [22], [23], was observed after seven days of laquinimod treatment. In contrast, we did not detect significant changes in the frequency of CD8^+^ cDC (Fig. S3). A similar pattern of DC subpopulations and T cells was observed in laquinimod-treated mice that had been immunized for EAE induction (data not shown). Further, we did not observe alterations in the frequencies of CD4^+^ or CD8^+^ T cells, showing that laquinimod treatment alone did not alter T cell homeostasis in naïve mice (Fig. 3A, Fig. S3). Interestingly, laquinimod treatment also did not alter proliferative responses in rats immunized with the encephalitogenic peptide, MBP p68-86 [24].

CD11b^+^Gr1^+^ cells, known as myeloid-derived suppressor cells, can exhibit regulatory function [25]–[27]. Upon laquinimod treatment, we observed a significant increase in frequency of CD11b^+^Gr1^+hi^ cells in naïve mice (Fig. 3B). As oral laquinimod treatment induced changes in both CD11c^+^ and CD11b^+^ APC subsets, we evaluated whether alteration of APC function was unique, or common to both subpopulations. Here, we evaluated whole splenic CD11b^+^, CD11b^+^CD11c^−^ and CD11c^+^ cells from laquinimod-treated mice. Under either proinflammatory-polarizing (Fig. 3C–E) or non-polarizing conditions (Fig. 3F), each of these myeloid APC subpopulations inhibited differentiation of Th1 and Th17 cells (Fig. 3C–F). An expansion in frequency of Treg was observed when we examined cultures using CD11b^+^ cells as APC (Fig. 3F). Our results demonstrate that T cell immune modulation induced by oral laquinimod treatment is not limited to its effects on one specific myeloid APC subset.

### Laquinimod induces type II monocytes and DC

**Figure 4:**
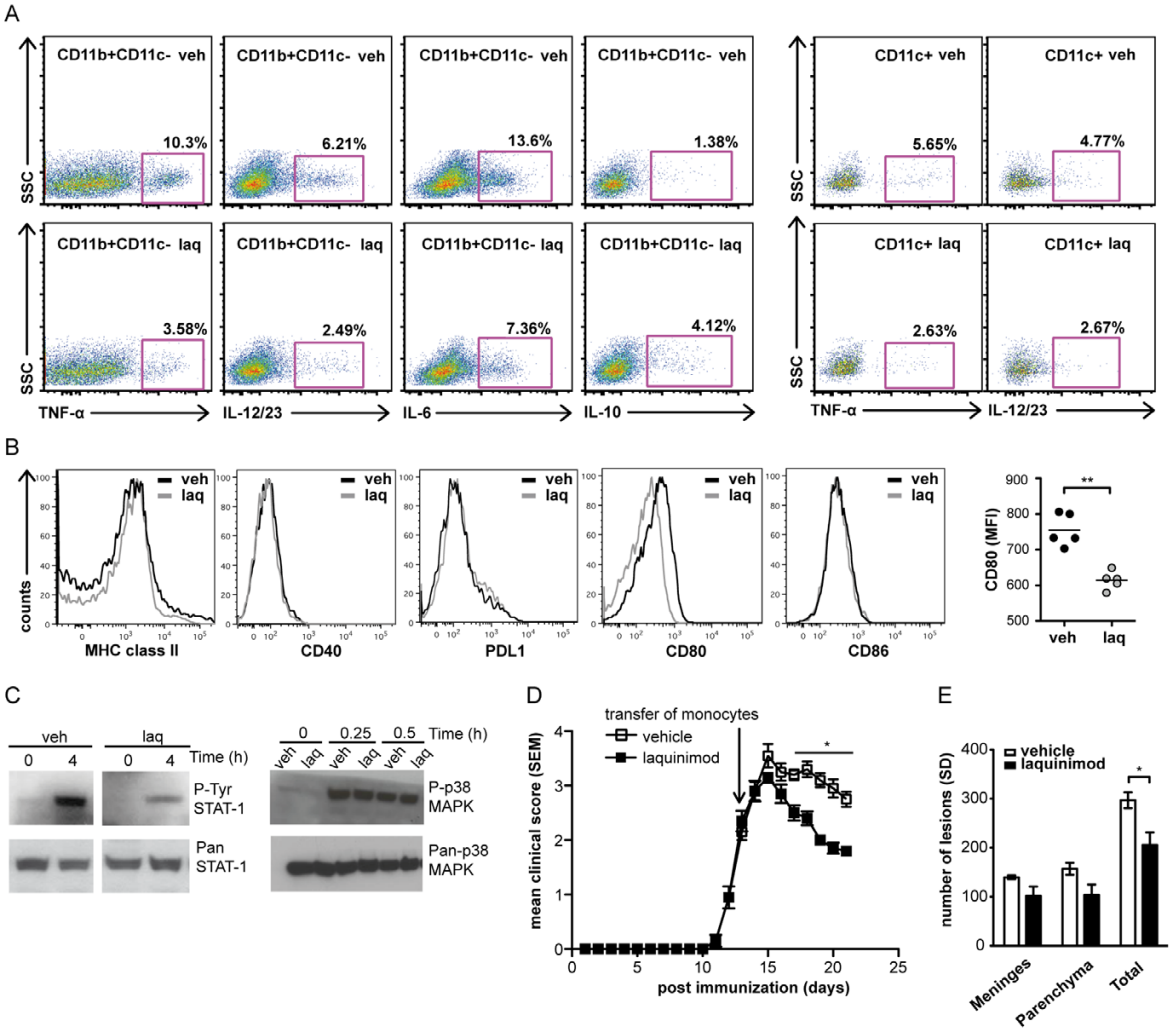
Laquinimod-induced type II (M2) monocytes reverse established EAE. Laquinimod-induced anti-inflammatory cytokine shift in CD11b^+^CD11c^−^ and CD11c^+^ cells. (A) FACS analysis of intracellular production of TNF, IL12/23p40, IL-6 and IL-10 by CD11b^+^CD11c^−^ and CD11c^+^ cells isolated from spleens of naive (unimmunized) mice treated with laquinimod or vehicle. (B) Cell surface FACS analysis of MHC II, proinflammatory and inhibitory costimulatory molecules on CD11b^+^CD11c^−^ cells. (C) In vivo laquinimod treatment affects signaling pathways that participate in proinflammatory cytokine production. Protein extracts were isolated from peritoneal macrophages of naïve C57BL/6 mice treated with laquinimod or vehicle and stimulated with LPS for various time points. Phosphorylated (P) STAT1, (P) p38-MAPK, Pan-STAT1 and Pan-p38-MAPK were detected by Western blot analysis. (D) CD11b^+^ cells from laquinimod-treated donor mice reversed established EAE. 5×10^6^ purified splenic CD11b^+^ cells from mice treated with laquinimod or vehicle were injected i.v. into recipient C57BL/6 mice immunized with MOG p35–55 after they developed a disease grade of 2 (black arrow indicates time point of adoptive transfer, (n = 5/group). (E) Quantification showed reduced total number of inflammatory foci after adoptive transfer of in vivo laquinimod treated CD11b^+^ cells into C57BL/6 mice immunized with MOG p35-55. Data shown in panels above are representative of three independent experiments. For EAE disease course, mean disease score ± s.e.m. are displayed; *P<0.05, **P<0.01 Mann-Whitney U test.

T cell immune modulation in glatiramer acetate (GA) therapy of MS and EAE is associated with expansion of anti-inflammatory type II (M2) monocytes that produce less proinflammatory cytokines and elevated levels of anti-inflammatory cytokines [16], [17], [28]. As myeloid APC in laquinimod treatment directed T cell immune modulation, we evaluated the cytokines produced by monocytes and DC. As shown in Figure 4A, both CD11b^+^CD11c^−^ monocytes and CD11c^+^ DC from laquinimod-treated naive (unimmunized) mice exhibited a type II phenotype, characterized by reduced cellular production of proinflammatory-polarizing cytokines IL-6, IL-12/23 (p40) and TNF, and a corresponding increase in anti-inflammatory IL-10. This type II cytokine profile was also detected in myeloid APC subsets isolated from laquinimod-treated mice immunized with MOG p35-55 (Fig. S4). We examined if laquinimod treatment alone influences expression of MHC II, CD40, CD80, CD86 and PD-L1, an inhibitory costimulatory molecule. Laquinimod treatment of naive mice was consistently associated with a small, but significant, reduction in CD80 (Fig. 4B). Our results clearly demonstrate that oral laquinimod treatment promotes development of both type II monocytes and type II DC, which are capable of inhibiting proinflammatory myelin-specific T cell responses.

As laquinimod inhibited proinflammatory cytokine production by monocytes and DC, we examined whether in vivo laquinimod treatment altered key signaling pathways that participate in expression of these molecules. Activation of the Janus kinase signal transducer and activator of transcription (JAK-STAT) pathway is required for expression of several proinflammatory cytokines of APC, thus influencing T-cell differentiation [29]. As shown in Figure 4C, LPS-induced STAT1 phosphorylation (activation) was reduced in CD11b^+^ monocytes isolated from laquinimod-treated mice. In contrast, in vivo laquinimod treatment did not alter phosphorylation of p38 mitogen-activated protein (MAP) kinase, representing another signaling cascade involved in stress responses. Thus, our data indicate that in vivo laquinimod treatment alters activation of select signaling pathways in myeloid type II APC.

In order to investigate whether laquinimod-induced type II myeloid APC could modulate clinical responses in vivo, we isolated type II CD11b^+^ monocytes from laquinimod-treated mice and adoptively transferred them into recipient mice with established EAE. These type II CD11b^+^ monocytes ameliorated clinical (Fig. 4D) and histological signs (Fig. 4E) of EAE in recipient mice.

## Discussion

In this report, we have observed that oral laquinimod treatment can prevent or reverse established EAE. In vivo laquinimod treatment was associated with alterations in myeloid APC subpopulations that included a reduction in CD4^+^ cDC, a potent DC subpopulation. We have shown that laquinimod treatment promoted development of anti-inflammatory type II monocytes and DC, reminiscent of our previous work demonstrating that in vivo treatment of mice with glatiramer acetate (GA, copolymer-1, Copaxone®), an approved therapy for RRMS [30], induced differentiation of anti-inflammatory type II monocytes [17]. Laquinimod did not alter numbers of CD4^+^ T cells. In a previous study, it was observed that laquinimod treatment reduced secretion of IFN-γ and IL-17 [9]. Here, we have demonstrated that oral laquinimod administration in EAE was associated with anti-inflammatory T cell polarization as demonstrated by reductions in frequencies of proinflammatory Th1 and Th17 cells in vivo, and an increase in Treg. By studying how in vivo laquinimod treatment influenced individual populations of myeloid APC or naive myelin-specific T cells, we demonstrated that T cell immune modulation was linked to induction of type II myeloid APC, but not from its effects on T cells alone. Thus, at physiologic levels achieved by in vivo treatment, laquinimod impacts APC, but may not influence T cells directly.

In general, CD4^+^ and CD8^+^ T cells, which express antigen-specific α/β^+^ T cell receptors, recognize peptide fragments of processed proteins only in association with polymorphic MHC molecules on APC [31]. In this regard, GA, which is a polypeptide-based therapy, provides antigenic determinants and leads to expansion of GA-reactive T cells that can be identified in therapy of MS [32]–[34] or EAE [17], [35], [36]. The requirement for MHC II on GA-induced type II monocytes was discovered at the time adoptive transfer of monocytes was first developed as an experimental paradigm to study how therapeutics can influence APC-T cell interaction in vivo [17]. In this regard, GA-induced type II monocytes from wild-type mice, but not from mice selectively deficient in MHC II, induced T cell immune modulation (i.e. expansion of Treg and Th2 cells) and reversed EAE in recipient mice. However, as a synthetic heterocyclic molecule, laquinimod itself is unlikely to serve as a target for T cell recognition. Use of the EAE model permits investigators to characterize how laquinimod can alter T cell responses that are elicited by direct immunization with myelin peptides or proteins, a situation not encountered in MS, a naturally occurring disease. Evaluating whether laquinimod treatment of MS modulates T cell function in the absence of active antigenic stimulation may be more challenging. Our observations that in vivo laquinimod treatment of unimmunized mice modified expression of myeloid subpopulations and APC function should focus attention on exploring the mechanism of action of laquinimod in MS therapy on cells of the innate immune system. Our demonstration that laquinimod has a principle effect on innate immunity provides mechanistic insight relevant to results from the two recent phase III clinical trials in RRMS that tested laquinimod (0.6 mg daily) and indicated that dose provided a more pronounced effect on disability progression than relapse rate reduction [37], [38]. Further, our findings also suggest that laquinimod could be beneficial in secondary progressive MS, a phase that involves chronic inflammation and neurodegeneration that is thought to be driven by innate immunity [39].

A recent study evaluated the potential role of brain-derived neurotrophic factor (BDNF) in laquinimod treatment [40]. A small, but significant increase in serum BDNF levels was detected in laquinimod-treated MS patients. These authors also evaluated the role of BDNF in laquinimod treatment of EAE. It is known that BDNF-deficient mice develop more severe chronic EAE [41]. They demonstrated that monocytes from laquinimod-treated donor wild-type mice, but not monocytes from laquinimod-treated BDNF-deficient mice or from untreated wild-type mice, ameliorated EAE in recipient mice. The authors concluded that the effects of laquinimod on monocytes are BDNF-dependent. However, they did not transfer untreated BDNF-deficient monocytes, and therefore did not distinguish the influence of the production, or the absence, of BDNF alone on monocyte function independent of laquinimod treatment. In order to attribute the effect of laquinimod to BDNF production by monocytes using this experimental approach, it is advantageous to not only compare laquinimod-treated and untreated wild-type monocytes to laquinimod-treated BDNF-deficient monocytes, but to simultaneously compare the adoptive transfer of untreated BDNF-deficient monocytes to both untreated wild-type monocytes and to laquinimod-treated BDNF-deficient monocytes.

In this investigation, we have identified cellular mechanisms that contribute to immune modulation by laquinimod, focusing on the interaction of myeloid APC and T cells. Type II monocyte differentiation was associated with reduced production of proinflammatory IL-6, IL-12/IL-23 (p40) and TNF, and increased production of anti-inflammatory IL-10. It is important to characterize the molecular pathway(s) utilized by laquinimod for this type II cytokine profile. Laquinimod is not known to have a well-defined target, although some in vitro data suggest that quinoline-3-carboxamides bind S100A9, a calcium binding protein [42] that influences cell signaling. Other results indicate that this class of molecules may alter NF-κB signaling [43]. We have begun evaluating the signaling events contributing to type II APC differentiation in monocytes/macrophages isolated from laquinimod-treated mice. First, we focused on activation of STAT1, a transcription factor that participates in expression of several proinflammatory cytokines [44], [45]. Laquinimod treatment suppressed inducible STAT1, but did not alter activation of p38 MAPK, another signaling pathway involved in expression of proinflammatory cytokines that can be regulated independently or coordinately with STAT1 [46]. Interestingly, inhibition of STAT1 and p38 MAPK signaling was observed in development of type II monocytes by GA [17] (N. Molnarfi and S.S. Zamvil, unpublished), suggesting that the signaling events modulated by GA and laquinimod during type II APC differentiation are not the same. Our observations represent only the initial steps in understanding how laquinimod influences intracellular signaling pathways in type II myeloid cell differentiation. Although we used both in vivo and ex vivo analyses to evaluate type II myeloid cells, laquinimod was only administered in vivo, which we believe more closely reflects the physiology of laquinimod treatment in MS. In contrast with previous studies [9], [11], [21], [40], we evaluated APC-T cell interaction, the interface between innate and adaptive immunity, primarily by in vivo laquinimod treatment in the absence of peptide immunization, and therefore obviating concern of adjuvant. Our findings in this report support evaluation of type II myeloid cells in laquinimod treatment of MS patients.

## Materials and Methods

### Mice

C57BL/6, DBA/1 and SJL/J female mice, 5 to 8 weeks of age, were purchased from Jackson Laboratories (Bar Harbor, MN, USA). MOG p35–55–specific TCR transgenic 2D2 mice were provided by V.K. Kuchroo (Harvard Medical School, Boston, MA).

### *Ethics* Statement

The experimental protocol adheres to guidelines for animal use in research set by the National Institutes of Health and was approved by the Office of Research, University of California San Francisco (UCSF) Institutional Animal Care and Use Committee (IACUC Approval AN081032-03B).

### Peptides

Mouse MOG p35-55 (MEVGWYRSPFSRVVHLYRNGK) and mouse PLP p139-151 (HSLAKWLGHPDKF) were synthesized by Auspep (Parkville, Australia). Recombinant rat MOG (rMOG 1-125) was synthesized by AnaSpec (Fremont, CA).

### EAE Induction

Seven to 10-week-old female C57BL/6, DBA/1 or SJL/J mice were injected subcutaneously with 50 µg MOG p35-55, 50 µg rMOG or 100 µg PLP p139-151, respectively, in complete Freund's adjuvant (DIFCO Laboratories, Detroit, MI). After immunization and 2 days later, mice received 200 ng (C57BL/6) or 100 ng (SJL/J) pertussis toxin intraperitoneally (i.p.). For adoptive transfer, donor SJL/J mice were immunized as described above and treated daily with laquinimod or vehicle. 10 days later, cells from draining lymph nodes and spleen were isolated, re-stimulated for 48 h (20 µg/ml PLP p139-151), and injected i.p. into naive SJL/J recipients (10^7^ cells per mouse). Animals were observed daily and clinical scores were assessed as follows: 0, no signs; 1, decreased tail tone; 2, mild monoparesis or paraparesis; 3, severe paraparesis; 4, paraplegia and/or quadraparesis; and 5, moribund or death. All experiments were carried out in accordance with guidelines prescribed by the Institutional Animal Care and Use Committee at the University of California, San Francisco.

### Laquinimod treatment

Laquinimod (TEVA Pharmaceuticals Industries, Ltd (Israel)) was dissolved in purified water and administered daily (25 mg/kg) by oral gavage. This dose was chosen as it was previously observed that lower doses were less effective [9]. Treatment started at the day of EAE induction or after first disease exacerbation. Control mice received a similar volume of vehicle (water) daily [42]. In other experiments (e.g., monocyte transfer, cytokine analysis, in vitro APC-T cell assays), mice were treated for 10 days with laquinimod or vehicle before isolation of specific cell subsets.

### Generation of Th1 and Th17 cells

CD4^+^CD62L^+^CD44^+^ naïve T cells were magnetically sorted from TCR-transgenic 2D2 mice (purity greater than 96%) using magnetic beads (Miltenyi Biotec, Auburn, CA) and stimulated with MOG p35-55 (20 µg/ml) in the presence of antigen-presenting cells (APC, e.g., MACS-sorted CD11b^+^, CD11c^+^ cells) at a T cell/APC ratio of 1∶5. Th17 differentiation was induced by addition of 3 ng/ml TGF-β, 20 ng/ml IL-23, 20 ng/ml IL-6 (R&D Systems, Minneapolis, MN). For Th1 cells, 10 ng/ml IL-12 (R&D) were added. Cells were harvested between days 3 and 4, and cytokine production was analyzed using a FACS Canto flow cytometer (BD, San Jose, CA).

### Monocyte isolation and co-culture with naïve T cells

Splenic CD11b^+^ and CD11c^+^ cells were separated from laquinimod or vehicle-treated mice using magnetic beads (Miltenyi). We evaluated monocyte and dendritic cell preparations for expression of CD11b, CD11c, B220, CD3 (BD Pharmingen, San Diego, CA). Purity of monocytes and dendritic cells used as APC was routinely greater than 95%. CD11b^+^, CD11b^+^CD11c^−^ or CD11c^+^ APC were co-cultured with T cells from 2D2 transgenic mice and their respective antigen for 3 days at a ratio of 25∶1.

### T cell proliferation

Purified CD11b^+^ cells from laquinimod- or vehicle-treated mice were cultured with naïve CD4^+^ cells isolated from laquinimod- or vehicle-treated 2D2 mice and antigen (MOG p35-55, 20 µg/ml). Cells were cultured in 96-well microtitre plates at a concentration of 0.25×10^6^ cells/ml. Culture medium consisted of RPMI 1640 supplemented with L-glutamine (2 mM), sodium pyruvate (1 mM), penicillin (100 U/ml), streptomycin (0.1 mg/ml), 2-mercaptoethanol (5×10^−5^ M) and 10% (v/v) fetal bovine serum. Cells were incubated for 48 h and pulsed for 18 h with 1 µCi per well of [^3^H]-thymidine before harvesting.

### Adoptive transfer of monocytes

Monocytes were isolated from the spleens of 8–10-week-old mice treated with laquinimod or vehicle for 10 days. Monocytes were enriched by magnetic cell sorting using CD11b^+^ magnetic beads (Miltenyi), resulting in a general purity of at least 95%. Recipient C57BL/6 mice were immunized with MOG p35–55, randomized at EAE score 2 and injected intravenously (i.v.) with 5×10^6^ laquinimod- or vehicle-treated monocytes.

### Isolation of CNS infiltrating mononuclear cells

Isolation of CNS infiltrating cells was performed as previously described [47]. Briefly, mice were perfused using PBS. CNS tissue was manually cut into small pieces and incubated for 20 min in Hank's Buffered Saline Solution containing collagenase. Homogenate was resuspended in 30% Percoll (Sigma) and underlain with 70% Percoll and centrifuged for 30 min. Cells were harvested from the resulting interface.

### Histopathology

Brains and spinal cords of mice were fixed in 10% neutral-buffered formalin, sectioned and stained with Luxol fast blue (LFB) and hematoxylin and eosin (H&E). Meningeal and parenchymal inflammatory lesions and areas of demyelination were quantified as previously described [48], [49].

### Isolation of peritoneal macrophages

C57BL/6 mice were treated for 10 days with laquinimod or vehicle. On day 7 of treatment, mice received i.p. injection of 1 ml thioglycollate (Becton Dickinson). Three days later, macrophages were harvested from mice by peritoneal lavage. Peritoneal macrophages were plated on Petri dishes in Dulbecco's modified Eagle's medium (supplemented with 10% heat-inactivated fetal bovine serum) for 1 hour at 37°C in an atmosphere of 5% CO_2_. After incubation, cells were washed three times with Hank's balanced salt solution to remove non-adherent cells, and equilibrated with Dulbecco's modified Eagle's medium that contained 10% fetal bovine serum. Cells were allowed to rest for 24 h before treatment with LPS.

### Western blot

Peritoneal macrophages isolated from mice treated with laquinimod or vehicle were stimulated with LPS (1 µg/ml) for various time points. Cell pellets were treated with ice-cold lysis buffer (50 mM Tris-HCl, pH 7.4, 1% NP-40, 0.25% sodium deoxycholate, 150 mM NaCl, 1 mM EDTA, 1 mM phenylmethylsulfonylfluoride (PMSF), 1 mM Na_3_VO_4_, 1 mM NaF) containing protease inhibitors and phosphatase inhibitors (Roche). Proteins were separated by SDS-PAGE and transferred onto nitrocellulose membranes (Amersham Biosciences) for subsequent immunoblotting with antibodies specific for phosphorylated STAT1, pan-STAT1, phosphorylated p38MAPK and p38 MAPK (Cell Signaling Technology, Danvers, MA).

### Flow cytometry

Single-cell suspensions were incubated with anti-CD16/CD32 (1∶100) to prevent nonspecific antibody binding, then stained with anti-CD4, -CD8, -CD62L, -CD44, -CD11c, -CD11b, -B220, Gr1 (Ly6C/G), -CD3, -MHCII, -CD40, -PDL-1, -CD80, and -CD86 (all 1∶100) (eBioscience, San Diego, CA). Intracellular cytokine production by CD4^+^ T cells and APC was analyzed by monitoring the expression of IFN-γ, IL-17, GM-CSF, IL-6, IL-12/23 (p40), TNF and IL-10 (1∶100) (eBioscience). Foxp3 staining was performed according to the manufacturer's protocol (eBioscience). For intracellular cytokine staining, T cells were stimulated with phorbol 12-myristate 13-acetate (PMA, 50 ng/ml) plus ionomycin (500 ng/ml) in the presence of GolgiStop (1 µl/ml) (BD, San Jose, CA). CD11b^+^ cells were stimulated with LPS (1 µg/ml) for 12 h in the presence of GolgiStop. Cells were analyzed by flow cytometry on a FACS Canto (BD).

### Statistical analysis

Data are shown as mean ± s.e.m or s.d. We examined significance between groups using the Mann-Whitney U test. A value of P≤0.05 was considered significant.

## Supporting Information

Figure S1 **Splenocytes and lymph node cells were isolated from PLP-immunized mice treated with laquinimod or vehicle for 10 days.** Cells were restimulated in vitro and analyzed by FACS for secretion of IFN-γ, IL-17, GM-CSF and expression of CD25 and Foxp3 by CD4^+^ cells at the day of transfer into naïve SJL/J recipients. Data are representative of two independent experiments.
(TIF)

Figure S2 **Splenic purified CD11b^+^ cells from laquinimod- or vehicle-treated mice were used as APC in co-culture with untreated naive CD4^+^ 2D2 T cells.** Conversely, naive 2D2 T cells were isolated from laquinimod- or vehicle-treated 2D2 mice and cultured with purified vehicle treated CD11b^+^ cells and Ag (MOG p35-55). Proliferative response of 2D2 cells is displayed as counts per minute (cpm) after [^3^H]-thymidine incorporation. Results are shown as means of triplicates ± s.e.m. Data are representative of three independent experiments.
(TIF)

Figure S3 **DC were isolated from the spleen and defined as CD11c^high^ CD8^+^ cDC (CD11b^−^CD4^−^).** Relative percentages of CD8^+^ DC and total splenic CD8^+^ T cells from laquinimod-treated compared to control mice is shown (n = 4). Results are shown as means ± s.e.m. Data are representative of two independent experiments.
(TIF)

Figure S4 **Laquinimod modulates cytokine profiles of CD11b^+^CD11c^−^ and CD11c^+^ cells.** FACS analysis of cytokines produced by CD11b^+^CD11c^−^ and CD11c^+^ cells isolated from spleen of mice on day 11 after immunization with MOG p35-55 and treated with laquinimod or vehicle. Shown are TNF, IL12/IL23p40, IL-6 and IL-10. Data are representative of two independent experiments.
(TIF)
