# Supplementary figures and images for: Laquinimod, a Quinoline-3-Carboxamide, Induces Type II Myeloid Cells That Modulate Central Nervous System Autoimmunity
Source: PLoS One. 2012 Mar 30;7(3):e33797. doi: 10.1371/journal.pone.0033797 (PMC3316495; doi:10.1371/journal.pone.0033797)

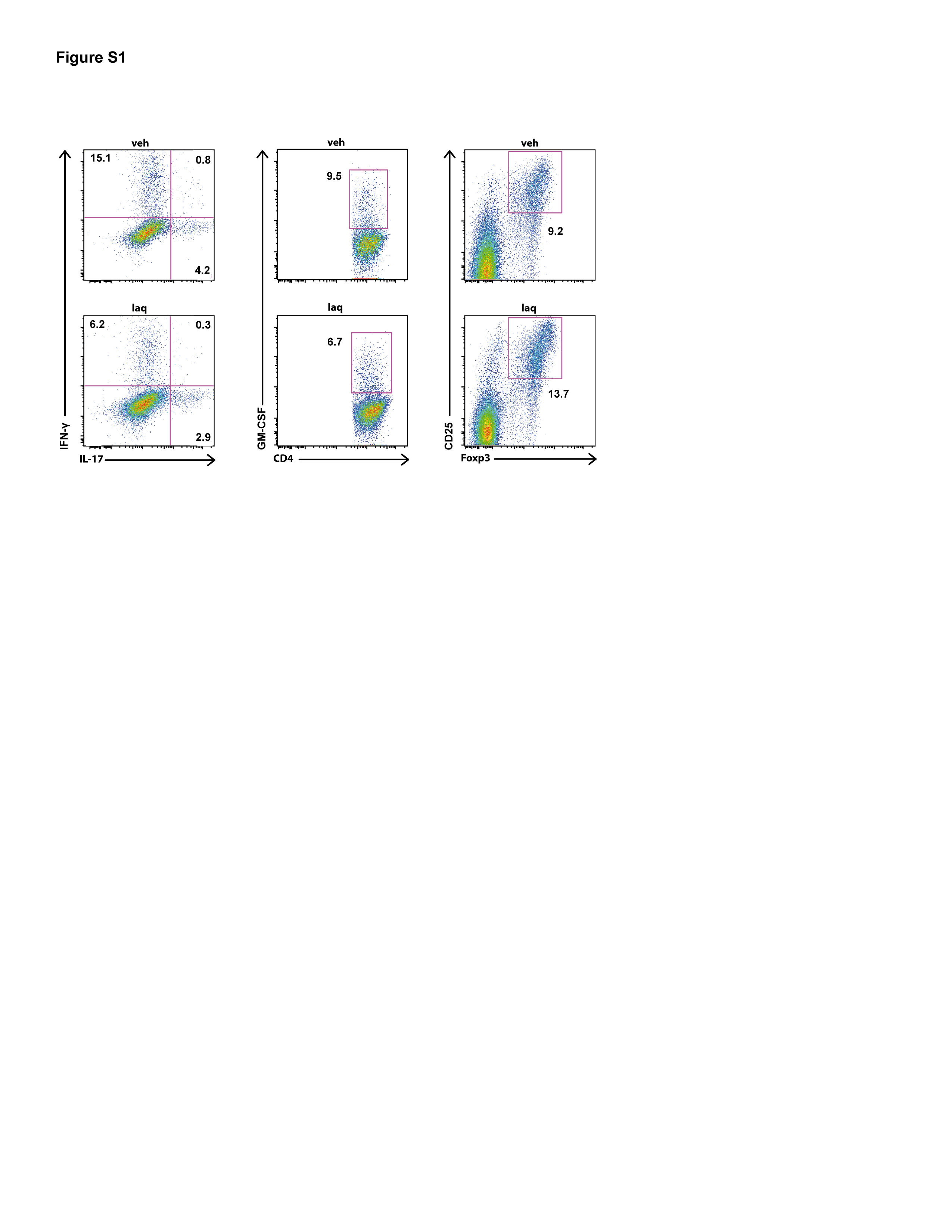

Supplement: Figure S1 — Splenocytes and lymph node cells were isolated from PLP-immunized mice treated with laquinimod or vehicle for 10 days. Cells were restimulated in vitro and analyzed by FACS for secretion of IFN-γ, IL-17, GM-CSF and expression of CD25 and Foxp3 by CD4+ cells at the day of transfer into naïve SJL/J recipients. Data are representative of two independent experiments. (TIF) [file pone.0033797.s001.tif]

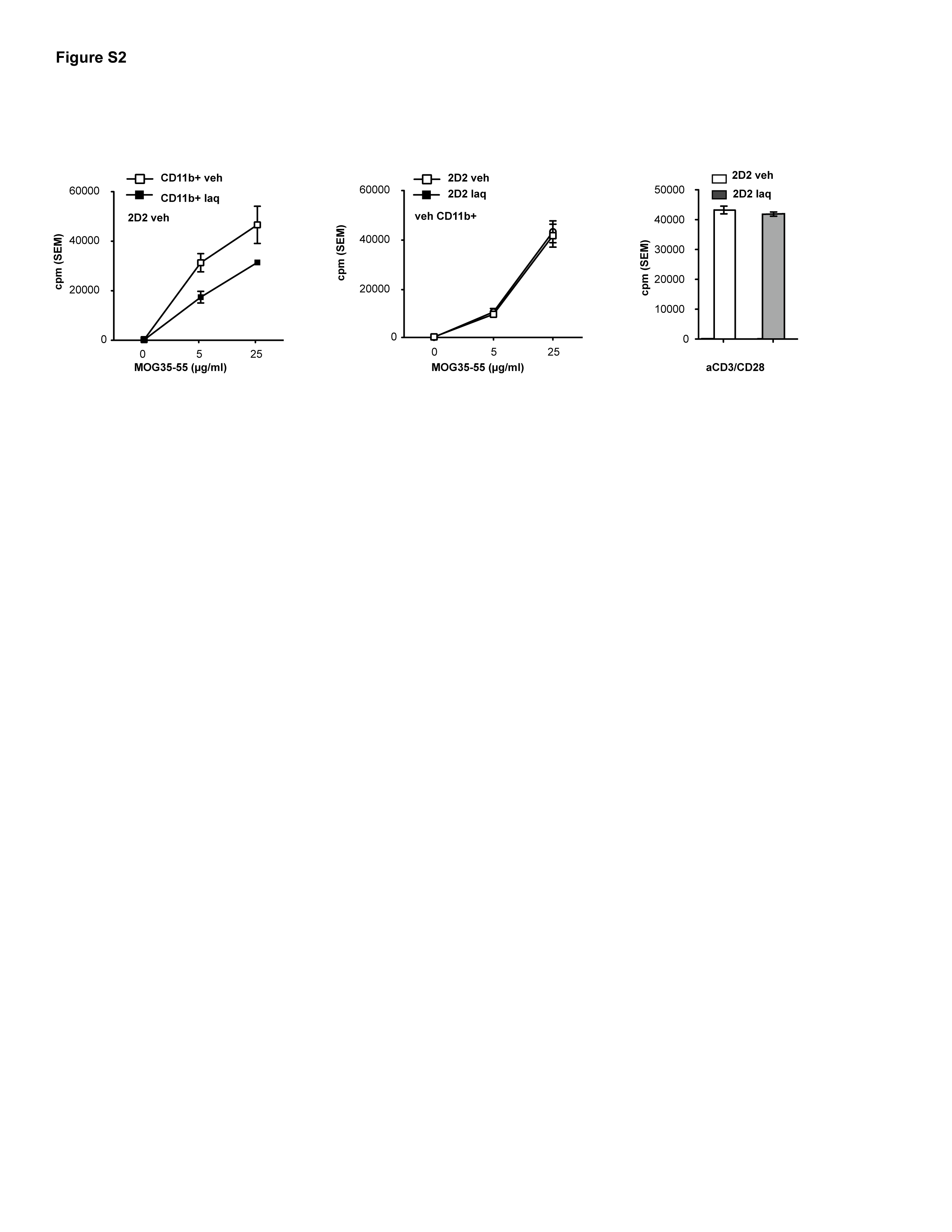

Supplement: Figure S2 — Splenic purified CD11b+ cells from laquinimod- or vehicle-treated mice were used as APC in co-culture with untreated naive CD4+ 2D2 T cells. Conversely, naive 2D2 T cells were isolated from laquinimod- or vehicle-treated 2D2 mice and cultured with purified vehicle treated CD11b+ cells and Ag (MOG p35-55). Proliferative response of 2D2 cells is displayed as counts per minute (cpm) after [3H]-thymidine incorporation. Results are shown as means of triplicates ± s.e.m. Data are representative of three independent experiments. (TIF) [file pone.0033797.s002.tif]

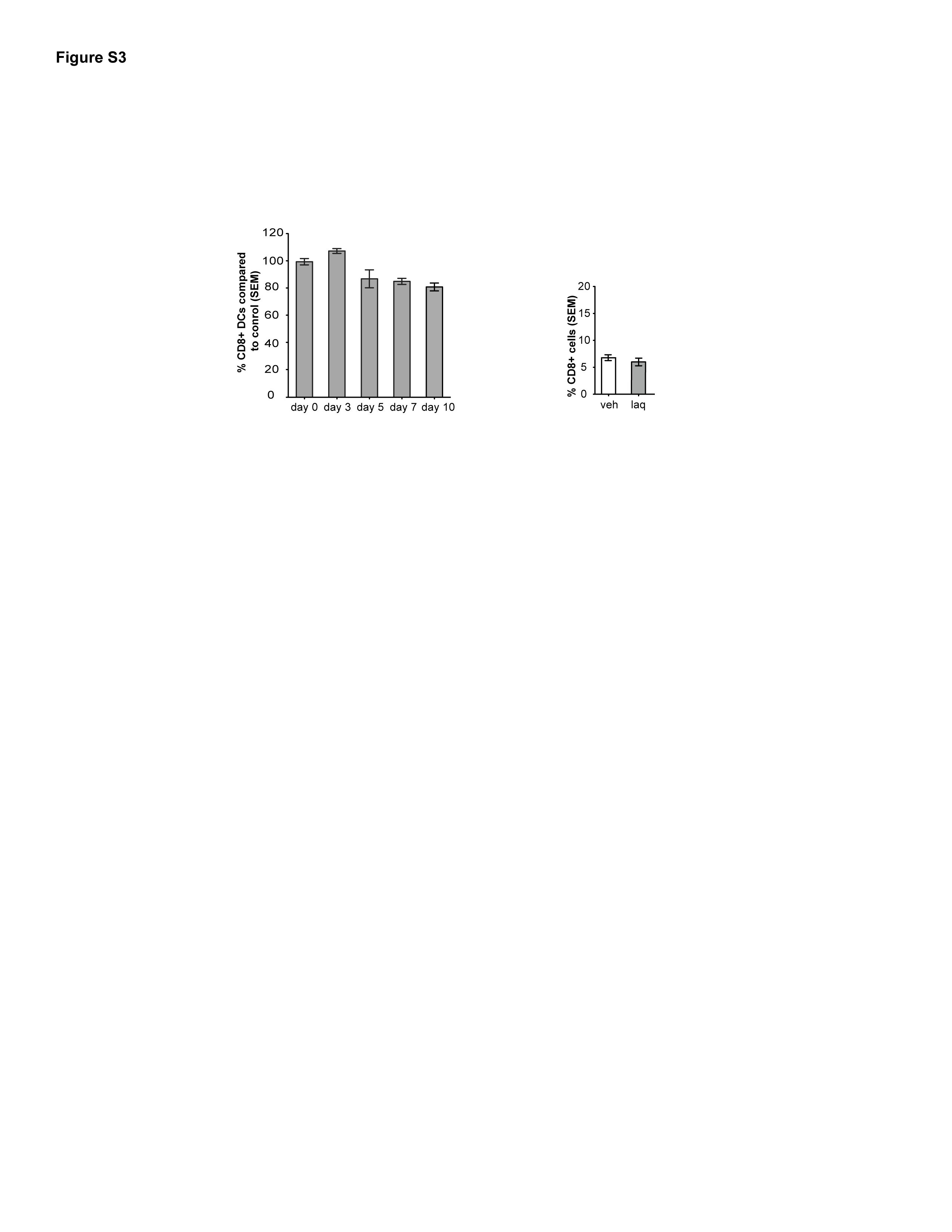

Supplement: Figure S3 — DC were isolated from the spleen and defined as CD11chigh CD8+ cDC (CD11b−CD4−). Relative percentages of CD8+ DC and total splenic CD8+ T cells from laquinimod-treated compared to control mice is shown (n = 4). Results are shown as means ± s.e.m. Data are representative of two independent experiments. (TIF) [file pone.0033797.s003.tif]

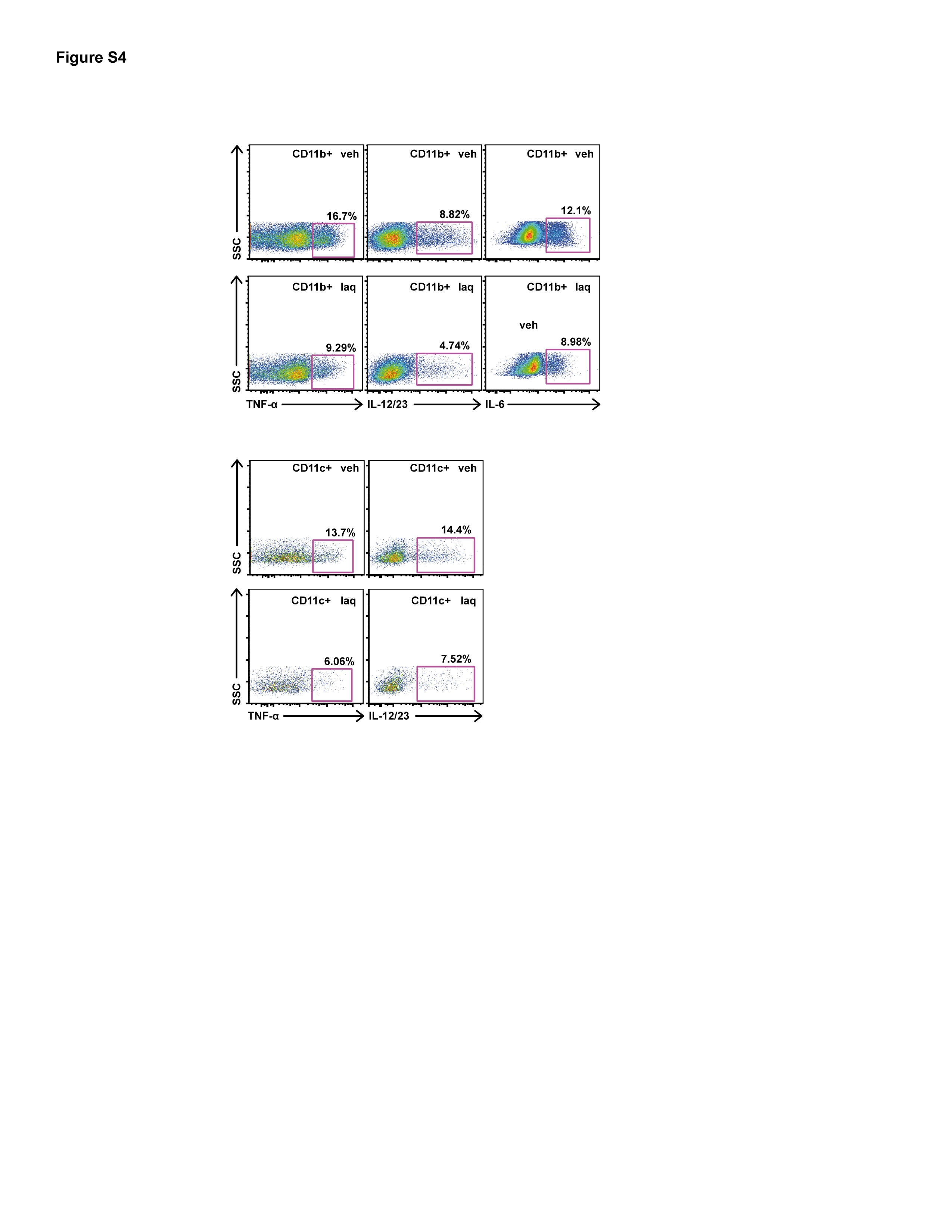

Supplement: Figure S4 — Laquinimod modulates cytokine profiles of CD11b+CD11c− and CD11c+ cells. FACS analysis of cytokines produced by CD11b+CD11c− and CD11c+ cells isolated from spleen of mice on day 11 after immunization with MOG p35-55 and treated with laquinimod or vehicle. Shown are TNF, IL12/IL23p40, IL-6 and IL-10. Data are representative of two independent experiments. (TIF) [file pone.0033797.s004.tif]
